# Anatomically correct visualization of the human upper airway using a high-speed long range optical coherence tomography system with an integrated positioning sensor

**DOI:** 10.1038/srep39443

**Published:** 2016-12-19

**Authors:** Joseph C. Jing, Lidek Chou, Erica Su, Brian J. F. Wong, Zhongping Chen

**Affiliations:** 1 Beckman Laser Institute, University of California, Irvine, 1002 Health Sciences Road, Irvine, CA 92617 USA; 2 Department of Biomedical Engineering, University of California Irvine, Irvine, CA 92697-2700 USA; 3 Department of Otolaryngology—Head and Neck Surgery, University of California, Irvine, 101 The City Drive South, Orange, CA 92868, USA.

## Abstract

The upper airway is a complex tissue structure that is prone to collapse. Current methods for studying airway obstruction are inadequate in safety, cost, or availability, such as CT or MRI, or only provide localized qualitative information such as flexible endoscopy. Long range optical coherence tomography (OCT) has been used to visualize the human airway *in vivo*, however the limited imaging range has prevented full delineation of the various shapes and sizes of the lumen. We present a new long range OCT system that integrates high speed imaging with a real-time position tracker to allow for the acquisition of an accurate 3D anatomical structure *in vivo*. The new system can achieve an imaging range of 30 mm at a frame rate of 200 Hz. The system is capable of generating a rapid and complete visualization and quantification of the airway, which can then be used in computational simulations to determine obstruction sites.

The nasal cavity, pharynx, and larynx are the anatomical structures that form the upper airway and are the conduits for the flow of air into the lungs. The upper airway has a complex shape and during respiration, pressure drops may lead to collapse and restriction of flow at any point from the palate to the epiglottis. Upper airway obstruction syndromes and related disorders are of profound importance to respiratory medicine, with obstructive sleep apnea being the most commonly known disease and linked to cardiovascular disease, stroke, and diabetes^1^. Knowing the anatomy of the upper airway in a given patient is of critical importance as it facilitates the design of patient-specific surgical therapy, as the sites and patterns of obstruction can be complicated and differ profoundly from patient to patient. However, it remains a challenge to obtain detailed structural upper airway anatomy without using imaging technologies that either utilize ionizing radiation such as computed tomography (CT) or are expensive such as magnetic resonance imaging (MRI), making neither appropriate as general screening measures. Moreover neither MRI nor CT are suitable for use in native sleep.

Optical techniques such as optical coherence tomography (OCT) can provide attractive solutions to this problem and have received significant attention and scrutiny. OCT is an optical interferometric imaging modality analogous to Ultrasound B-Mode imaging that acquires high-resolution micrometer scale cross-sectional images of living tissues. Endoscopic based OCT has utilized miniature scanning fiber optic probes to enable three dimensional imaging of coronary vasculature^2^,^3^, gastrointestinal tract^4^,^5^, and lower airway features^6^,^7^. Here the organs of interest are either relatively small or known to be a fixed distance away with a more or less cylindrical shape, such that the lumen walls under study are consistently positioned within the imaging range of OCT, which is generally limited to 6–8 mm. For larger organs, anatomical or long range OCT (LR-OCT) was developed as an offshoot of standard endoscopic OCT imaging with the primary focus on increasing the imaging range of OCT at the possible cost of speed or resolution. This facilitates quantitative optical ranging to acquire the size and shape of various hollow organs^8^,^9^,^10^,^11^,^12^. In the airway LR-OCT is a compelling approach as thin, fiber optic probes can be easily inserted to perform volumetric imaging of the lumen walls. OCT anatomical measurements of airway feature sizes have been shown to be accurate with a mean difference of 0.5% in comparison with CT scans^11^. More recent LR-OCT based systems have been developed featuring improved imaging range, speed, and sensitivity as compared to the first anatomic OCT systems and have been used to demonstrate the feasibility of human upper airway imaging *in vivo*^13^,^14^,^15^,^16^. Despite these reports the current systems technologies are still limited by inadequate imaging range and relatively slow data acquisition speeds. In the airway, the maximum cross-sectional diameter can reach up to 5 cm depending upon the phase of the respiratory cycle, and these distances are beyond the capabilities of previous reported systems which have imaging ranges around 12–15 mm^13^,^14^. Without this imaging range, full structural anatomic imaging of the upper airway cannot be accomplished. In addition, the acquired OCT data from these systems do not provide information on the bending of the airway. Full quantification of the airway shape requires a position sensor to be integrated with the OCT probe.

In this paper, we report a new LR-OCT system that integrates high speed imaging with real-time position tracking to generate an accurate 3D anatomical structural model of the human upper airway. The high speed LR-OCT is based on a wavelength sweeping vertical cavity surface emitting laser (VCSEL) swept source, which offers many attractive attributes for long-range based OCT imaging^5^,^17^,^18^. These lasers feature a much longer coherence length and higher repetition rate compared with other OCT laser sources, which allows for vastly increased imaging range and imaging speed without the penalty of reduced resolution or sensitivity. We have adapted a VCSEL source into our LR-OCT imaging system in order to achieve full imaging coverage of the upper airway. An imaging range of 30 mm was achieved allowing for visualization of airway diameters of greater than 50 mm. A high speed endoscopic imaging probe was developed to acquire *in vivo* upper airway images at 200 frames per second. The imaging probe incorporated a position sensor to record the location of each cross-sectional scan, which is crucial for the generation of three dimensional models of the complex upper airway shape. Using these structural models, computational simulations can be performed to provide physicians a better understanding of airflow within the upper airway^19^,^20^,^21^,^22^. The first high speed *in vivo* imaging of human upper airway with integrated position tracking was demonstrated.

## Results

### OCT System Design

**Figure 1:**
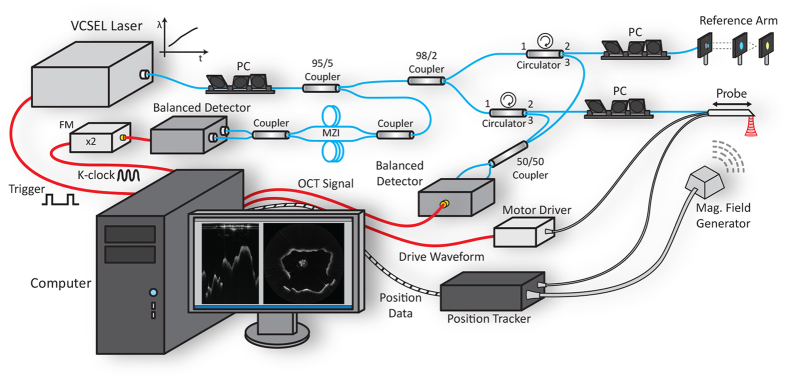
OCT System Schematic.

Our imaging system is based on Fourier domain OCT and utilizes a 100 kHz VCSEL source to perform high speed long range imaging of the airway lumen. In our previous designs, we utilized acousto-optic modulators (AOMs) to generate a heterodyne detection scheme, which allowed for imaging across the full coherence length of the laser. However, AOMs are dispersive and narrow bandwidth devices that are subject to signal loss and thus their use results in reduction in desirable OCT system properties such as sensitivity and axial resolution. VCSEL sources rely on their ultrashort cavity length to generate a very narrow instantaneous line width which enables an inherently long imaging range, thus in turn also greatly simplifying the optical system design and digital signal processing^17^. Our OCT system (Fig. 1) utilizes a Mach-Zehnder based interferometer setup with a 99:1 fiber coupler splitting the light between an optical delay line in the reference arm and the imaging probe in the sample arm. Dual circulators in both the reference and sample arms direct the back scattered light to a 50:50 coupler at which point the resulting OCT interference fringe is detected utilizing a 1.6 GHz wide balanced photodetector and sampled using a high speed 1.8 GHz digitizer.

Proper Fourier domain image processing requires the OCT interference fringe to be linearly sampled in wavenumber or K-space which is generally achieved by a calibration algorithm or through nonlinear time sampling using a K-clock. The VCSEL source offers a K-clock which provides for an imaging range of up to 12 mm in air, however to ensure visualization of the entire airway lumen, we generated an external K-clock signal by building a separate Mach-Zehnder interferometer using dual 50:50 couplers with a path length difference of 15 mm between the two arms^23^. This K-clock interferogram was detected using a second 1.6 GHz balanced photodetector, frequency doubled and amplified before being fed into the external clock port of the digitizer which allowed for a 3 dB imaging range of 30 mm. Imaging was performed at 200 Hz with one frame being comprised of 500 A-scans and GPU based processing was utilized to enable real time display of the acquired LR-OCT frames.

### OCT Imaging Probe

**Figure 2:**
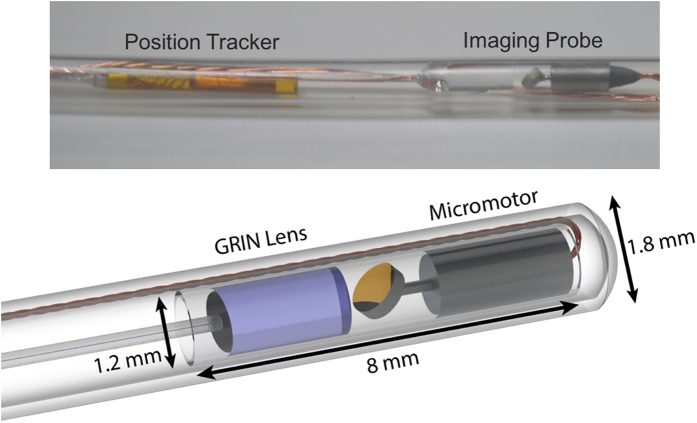
Fully integrated OCT imaging probe and position tracker (top). Schematic rendering of optical assembly (bottom).

Rotational scanning in endoscopic OCT imaging can be achieved either through proximal or distal design schemes where in the former the entire probe body is rotated by an external motor while the latter utilizes a single rotational element at the tip of the probe to provide scanning. For high speed imaging of large lumen diameters such as in the airway, distal design schemes offer better stability during scanning since the lumen wall does not fully constrain the probe body and provide stability from vibrational motion during scanning such as in cardiovascular imaging. We developed a long range based OCT imaging catheter (Fig. 2) using a commercial high speed micromotor. Both the micromotor and the focusing gradient index (GRIN) lens featured an outer diameter (OD) of 1 mm and were positioned within an 8 mm long, 1.2 mm OD glass capillary. A 0.9 mm diameter micromirror was positioned onto the shaft of the motor which was cut with a 45° face to direct light out laterally into the lumen walls. Imaging was performed at 200 frames per second while volumetric data was acquired by using an external motorized translation stage to retract the probe from the airway at a speed of 10 mm/s. The entire probe body was contained within a sealed plastic polymer sheath of 1.8 mm OD, which facilitated sterility of the probe as well as structural support for insertion of the probe into the airway.

### Position Tracking

Position tracking of the probe during image pullback was acquired using a commercial electromagnetic tracking system comprised of an external magnetic field generator and sensor (Ascension Technology Corp, VT, USA). The field generator was placed adjacent to the individual while the field sensor featured an OD of 0.9 mm which allowed it to be fully integrated together with the OCT imaging probe instead of being placed externally as has been reported in the past. The sensor was positioned with an offset of 6 mm from the micromotor housing to reduce electromagnetic interference generated by the motor during imaging. During imaging, a set of 3D coordinates was acquired along with each OCT cross-section to generate a pullback travel path which was then used to reconstruct the anatomically correct OCT volumetric model.

### Imaging

**Figure 3:**
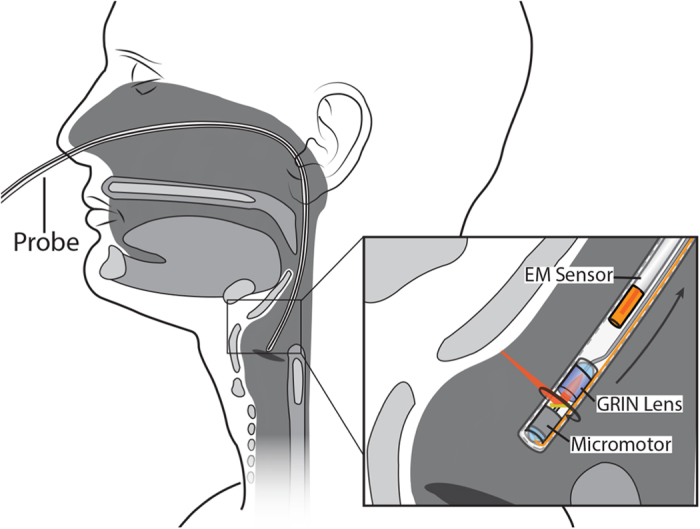
Diagram of LR-OCT imaging of Upper Airway.

**Figure 4:**
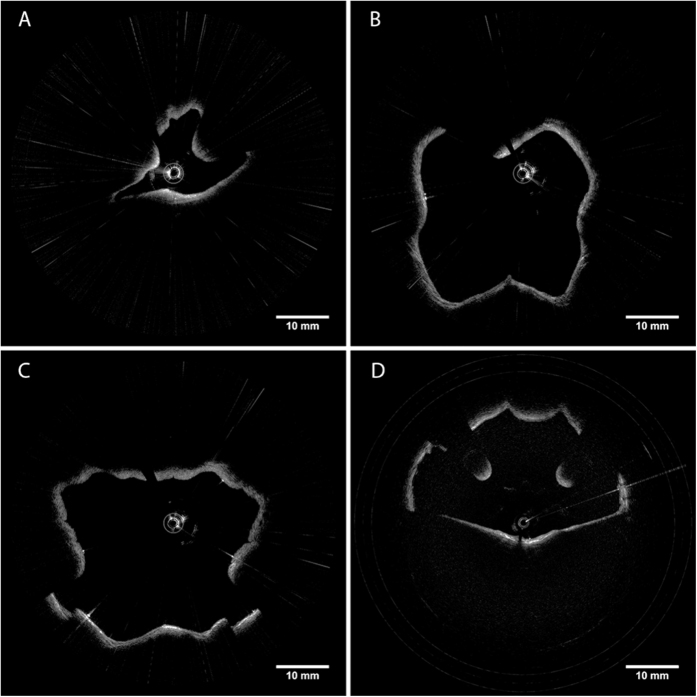
OCT images of upper airway acquired using VCSEL source. (**A**) Left nostril (nasal cavity), (**B**) Choana (nasopharynx), (**C**) Base of tongue (oropharynx), (**D**) Epiglottis (hypopharynx).

All studies were done under the approval of the UC Irvine institutional review board (IRB). Imaging was conducted in accordance with guidelines set forth in protocol number 2003–3025 and with individual informed patient consent. Patients and volunteers were locally anesthetized and decongested with a 4% lidocaine/oxymetazonline HCl nasal spray. After approximately 5 minutes when the nasal cavity was reported to be numb, the imaging probe was inserted into the nose and guided through the nasopharynx and oropharynx down to the top of the pyriform sinus using patient feedback as well as OCT imaging to verify positioning. The probe was then retracted from the airway while performing simultaneous OCT imaging of the lumen wall and position tracking of the distal tip of the probe which lasted approximately 8–10 seconds (Fig. 3). Figure 4 shows cross-sectional slices of the varied geometries found within an adult upper airway which requires the use of long imaging range system to capture. Full coverage over the entire airway lumen is possible with gaps in the lumen surface limited only by blockages of line of sight.

### Model Generation

Image segmentation was first performed using a semi-automated edge detection scheme on each acquired OCT image set to generate a preliminary measure of the airway lumen surface^24^. Spline based curve fitting was then applied over regions where breaks in the lumen surface occurred, such as the area obscured by the motor wire in the probe. The segmented cross sections were then aligned orthogonally to the acquired positional tracking path to generate an anatomically correct image stack using a custom developed MATLAB script. From this stack, a 3D mesh and surface model of the airway lumen was generated (Supplemental Video) using a combination of commercial and free software packages.

### Computational Simulations

Computational fluid dynamics (CFD) was performed on the 3D surface model using previously described methods^20^. Briefly, CFD results were calculated using a custom developed 3-dimensional solver based on the direct numerical simulation lattice Boltzmann methods (DNS-LBM). Compared to conventional DNS approaches which solve the 3-dimensional Navier-Stokes equations (DNS-NS), the DNS-LBM equations are simpler to solve and more conducive to parallelization for faster calculation times. Moreover, in DNS-LBM, pressure is a local property which can be derived directly, where in DNS-NS, pressure calculations require the computational complexity of solving additional elliptic Poisson equations. Utilization of spatial pressure and velocity gradients have been shown to correlate well with identifying obstruction locations^20^,^22^.

## Discussion

Upper airway obstruction can often have a complex multifactorial origin which is difficult to pinpoint. Surgical treatment for improving airway obstructions can span the spectrum from simple tonsillar fossa operations to major craniofacial reconstruction with success rates entirely dependent upon the correct identification of the site(s) of obstruction. Therefore it is highly important for the airway to be quantitatively studied as a complete structure. Both MRI and CT imaging have been used to generate full volumetric models of the upper airway for the study of airway obstructions^25^,^26^,^27^,^28^, however neither are particularly practical as standard screening measures. MRI can provide dynamic high resolution and high contrast visualizations of the airway lumen but the exceedingly high cost per study and limited accessibility precludes its adoption for widespread airway imaging. In addition, sedation may be required to prevent motion artifacts which may impact muscular elements from maintaining airway patency. CT can perform rapid volumetric imaging of the entire airway lumen but poses the risk of exposure to ionizing radiation especially during longer dynamic acquisitions. We have demonstrated the feasibility to acquire a complete quantitative measure of upper airway shape and size using a VCSEL based LR-OCT system which is both minimally invasive and free of ionizing radiation. In addition, our LR-OCT system is well suited for imaging in the office as well as potentially the sleep laboratory where polysomnography is performed in overnight sleep studies. The size of our probe also allows for integration with conventional endoscopes by inserting the probe into the working channel of the scope and thus increasing physician familiarity and adaptability.

**Figure 5:**
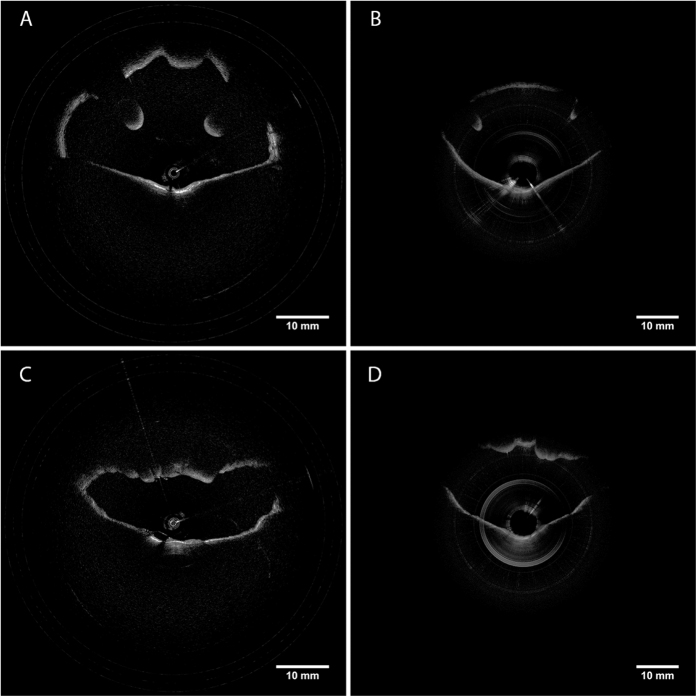
Comparison of OCT images from new VCSEL based system (**A**,**C**) and previous LR-OCT (**B**,**D**) demonstrating improved imaging range (**A**,**B**) Hypopharynx, (**C**,**D**) Oropharynx.

Our VCSEL based long range system has achieved many advancements over our previous systems. Our system imaging range has improved from 13 mm up to 30 mm which greatly improves coverage of the entire airway lumen. Figure 5 shows a comparison between OCT images acquired with our new VCSEL LR-OCT system and our previous system and demonstrates the importance of this increased imaging range in upper airway imaging. During imaging, the probe is often not centrally positioned but instead is pushed up against the lumen wall. With our previous design, this situation would lead to a lack of complete visualization of portions of the airway such as in Fig. 5b and d where the epiglottis and lateral pharyngeal walls of the airway become obscured. The improved imaging range of our new LR-OCT system allows us to visualize the complete airway even in these situations with gaps in coverage of the airway contour limited only to regions blocked from line of sight. Currently, the only major region obscured from visualization is the epiglottic vallecula which is the region behind the epiglottis. However, this region generally does not contribute to airway obstruction since it is surrounded by rigid cartilage. The complete visualization of the lumen shape also allows for quantitative measurements of airway dimensions. Cross-sectional area can be computed through the numerical integration of the wall distance of each A-line in an OCT slice. Volume of the scanned airway is determined by measuring the total enclosed space within the tracker based reconstructed 3D mesh. Our imaging speed as compared to our previous system has likewise increased from 50 to 200 frames per second. This improved speed has allowed for reduced procedure times which minimizes patient discomfort during imaging. In addition, faster imaging speeds opens the door for analyzing dynamic measurements of the airway such as sleep related collapse^29^ or lumen wall compliance^30^,^31^.

**Figure 6:**
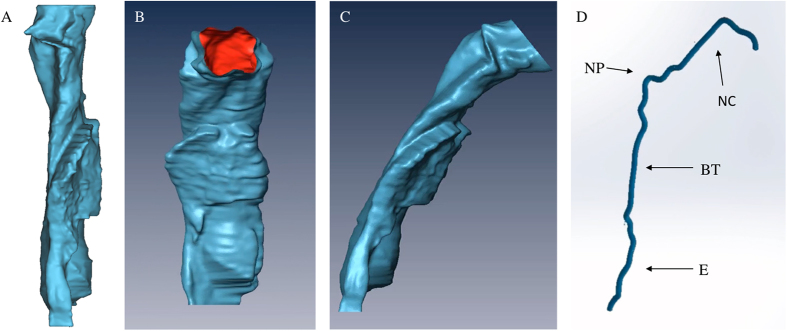
Reconstructed model without tracking data (**A**). Anterior (**B**) and Lateral (**C**) view of reconstructed 3D OCT data based on probe position data (**D**). Nasal Cavity (NC), Nasal Pharynx (NP), Base of Tongue (BT), Epiglottis (**E**).

**Figure 7:**
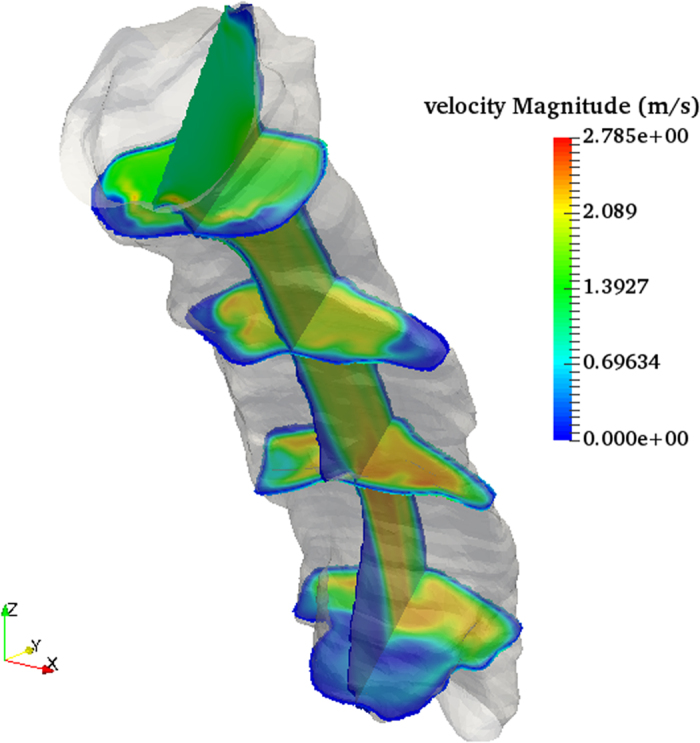
CFD simulation of airflow within generated 3D OCT volume using lattice Boltzmann method showing laminar flow within unobstructed airway. Inflow of 9.86 L/min.

We have also developed, to the best of our knowledge, the first fully integrated endoscopic OCT imaging probe with simultaneous position tracking for use in the airway *in situ*. Position tracking data is a crucial step for airway modeling as without it the resulting image stack would take on a cylindrical form which grossly misrepresents the airway shape. Previous reports^9^,^13^ with position sensing either utilized a side by side design with the tracking sensor external to the actual imaging probe or acquired OCT and position data sequentially. The first design necessitates a larger overall probe size and increases the difficulty of insertion into the airway. The second approach requires leaving the probe sheath in place in the airway and performing two pullbacks to acquire OCT and tracker data with the assumption that the shape of the sheath inside the airway does not change. By incorporating the position sensor within our probe design, we are able to minimize the overall size of our probe while still being able to record the location of each image to within a single pullback. Figure 6a shows a reconstructed model built without position data in which each slice is directly stacked on top of the last and bears little resemblance to actual upper airway shape. Figure 6b and c show the same model but reconstructed using the simultaneously acquired position data shown in Fig. 6d. The transverse travel path features an S shaped form with three predominate curvature locations. The very first curve at the top of the path is from the traversal through the nasal cavity which we exclude in our models since the majority of airway obstructions occur within the velopharynx and oropharynx. The second major bend occurs in the transition between the nasopharynx and the oropharynx causing the probe to be pressed against the posterior wall of the airway. The last major bend is an inverse curve caused by the emergence of the epiglottis which forces the probe to be positioned against the anterior wall of the laryngopharynx. With an accurate reconstruction of airway shape, computational fluid dynamics simulations (CFD) can be used to estimate flow velocity and pressure distributions and gain a more comprehensive understanding of the origins of airway obstruction. Figure 7 shows a direct numerical simulation using lattice Boltzmann methods on an unobstructed airway model with expected high laminar flow and matches previously reported simulated results on reconstructed models from CT data^19^,^20^.

We have demonstrated the ability to fully visualize the shape and size of the upper airway using a VCSEL based LR-OCT system with an integrated positioning sensor. This approach offers a rapid, non-ionizing, and minimally invasive means to generate accurate 3D reconstructions of the upper airway that is also suitable for in clinic diagnostics. Using these modes, CFD simulations can be performed to provide important information about airflow dynamics to aid in the identification of obstruction locations. Using this information together with structural measurements of airway size, clinicians will be able to better design and target specific regions in the airway for the treatment of obstruction.

## Additional Information

**How to cite this article**: Jing, J. C. *et al*. Anatomically correct visualization of the human upper airway using a high-speed long range optical coherence tomography system with an integrated positioning sensor. *Sci. Rep.* **6**, 39443; doi: 10.1038/srep39443 (2016).

**Publisher's note:** Springer Nature remains neutral with regard to jurisdictional claims in published maps and institutional affiliations.

## Supplementary Material

Supplementary Information

Supplementary Video
