# Supplementary material for: Anatomically correct visualization of the human upper airway using a high-speed long range optical coherence tomography system with an integrated positioning sensor
Source: Sci Rep. 2016 Dec 19;6:39443. doi: 10.1038/srep39443 (PMC5171831; doi:10.1038/srep39443)
Supplement: Supplementary Information [file srep39443-s1.pdf]

**Anatomically correct visualization of the human upper airway using a high-speed long range optical coherence tomography system with an integrated positioning sensor**

Joseph C. Jing[1,2], Lidek Chou[1], Erica Su[1], Brian J.F. Wong[1,2,3], Zhongping Chen[1,2,*]

[1]Beckman Laser Institute, University of California Irvine
[2]Department of Biomedical Engineering, University of California Irvine
[3]Department of Otolaryngology—Head and Neck Surgery, University of California Irvine

*Corresponding author:

Zhongping Chen, PhD
Professor
Dept. of Biomedical Engineering
University of California, Irvine
Irvine, CA 92617
e-mail: z2chen@uci.edu

# Video Legend

Supplementary video: Reconstructed 3D surface model of an adult upper airway lumen from acquired long range OCT images.
